# Colonic Diffuse Large B-Cell Lymphoma in a Liver Transplant Patient with Historically Very Low Tacrolimus Levels

**DOI:** 10.1155/2012/952359

**Published:** 2012-07-29

**Authors:** Christopher M. Moore, Ihab Lamzabi, Anne K. Bartels, Shriram Jakate, David H. Van Thiel

**Affiliations:** ^1^Section of Hepatology, Rush University Medical Center, Chicago, IL 60612, USA; ^2^Section of Pathology, Rush University Medical Center, Chicago, IL 60612, USA

## Abstract

Posttransplant lymphoproliferative disorders (PTLDs) comprise a wide spectrum of hematologic malignancies that are found increasingly in orthotopic liver transplant (OLT) patients given the rising frequency of these surgeries and their long-term success. PTLDs are highly correlated with both the Epstein-Barr virus (EBV) infection and the degree of immunosuppression involved. Herein is reported a case of a 53-year-old male with successfully treated hepatitis C virus genotype 4 and hepatocellular carcinoma who underwent OLT and developed symptoms of weakness and poor appetite 4 years later while on tacrolimus 3 mg b.i.d. with historically very low plasma levels. He was found to be anemic and colonoscopy revealed a 4.5 cm cecal diffuse large B-cell lymphoma (DLBCL). Further workup revealed mesenteric lymph node enlargement consistent and nodal DLBCL dissemination. He was treated with cyclophosphamide-hydroxyldaunorubicin-oncovin-prednisone-rituximab (CHOP-R) chemotherapy and his tacrolimus dose was lowered. Additionally, he manifested PTLD-associated cryoglobulinemia leading to acute kidney injury. After a prolonged hospitalization he was discharged with close followup.

## 1. Introduction

Orthotopic liver transplantation (OLT) is a definitive therapy for fulminant liver failure and advanced chronic liver disease including hepatitis C virus (HCV) and alcoholic cirrhosis [1]. OLT incurs the cost of chronic immunosuppressive therapy-associated renal injury and posttransplant lymphoproliferative disorders (PTLDs) [2]. With the increased frequency of OLT and prolonged survival of these patients through (1) better patient allocation, (2) improvement in both surgical technique, and (3) post-OLT immunosuppressive therapies, the concern for and treatment of PTLD has increased.

PTLDs are a diverse group of potentially lethal neoplasms whose incidence is 1.2% for all solid organ transplants and 0.9% for OLT specifically [2]. PTLDs are highly correlated, and distinguished from lymphomas, by the association with Epstein-Barr virus (EBV) infection, the duration and intensity of immunosuppressive therapies, and type of organ transplanted [3]. Reduction in immunosuppressive therapy may lead to regression in a proportion of cases whereas others may progress to lymphoma [4]. 

Depending upon the initial site of the PTLD, the clinical presentation can range from lymphadenopathy and “B-symptoms” to renal failure and gastrointestinal hemorrhage and/or obstruction. The treatment is based upon location, extent, and metastasis and can include surgery, radiation, or chemotherapy [3]. 

Herein is a case report of an OLT patient with colonic PTLD diffuse large B-cell lymphoma (DLBCL) presenting with symptomatic anemia and cryoglobulin-induced acute kidney injury (AKI).

## 2. Case Presentation

A 53-year-old Egyptian male with HCV genotype 4 cirrhosis and hepatocellular carcinoma (HCC), who was successfully treated with antiviral HCV therapy prior to OLT in 2007, was admitted to the hospital in February 2012 for increasing weakness and poor appetite in the past month. His comorbid diseases included diabetes mellitus and chronic kidney disease (CKD). His immunosuppressive regimen was tacrolimus 3 mg bid, at a stable dose, with an admission plasma level of 3.1 ng/mL. He was noted to have had both chronically normal liver injury tests and very low tacrolimus plasma levels (<5 ng/mL) during outpatient visits.

Upon presentation his blood laboratory specimens revealed a number of abnormalities (see Table 1). His hemoglobin (hgb) levels had decreased from 9.7 (baseline) to 7.6 g/dL. His WBC was 3.5 × 10^3^/*μ*L with a 60% lymphocyte differential. He had denied any overt bleeding in recent memory and was not on any anticoagulants. Notably his creatinine (Cr) levels had risen from a baseline of approximately 2 to 4.9 mg/dL upon admission; he was still producing urine with noted proteinuria (~7 g/day) and hematuria. Ultrasound of the kidneys was consistent with CKD. On day 7 he underwent a kidney biopsy displaying refractile eosinophilic hyaline globules in glomerular capillary lumen suggestive of cryoglobulin deposits. Serum cryoglobulin precipitate levels for IgG were 122 mg/dL, IgM 113 mg/dL, IgA < 1.0 mg/dL, and RF > 650 IU/mL. His Cr at that time was 4.3 mg/dL, having received daily IV fluids in the interval. He was started on plasmapharesis (to receive six total treatments during this hospitalization) and oral prednisone starting on hospital day 8, to continue until discharge home. His liver-injury parameters including total bilirubin (TB), alkaline phosphatase (AP), and transaminases were normal, as was the INR upon admission and it remained so throughout the hospitalization. A liver biopsy on day 7 displayed no signs of viral inclusions, immune rejection, nor signs of fibrosis or lymphoma. Serum HCV-RNA was less than 12 IU/mL at admission.

**Figure 1:**
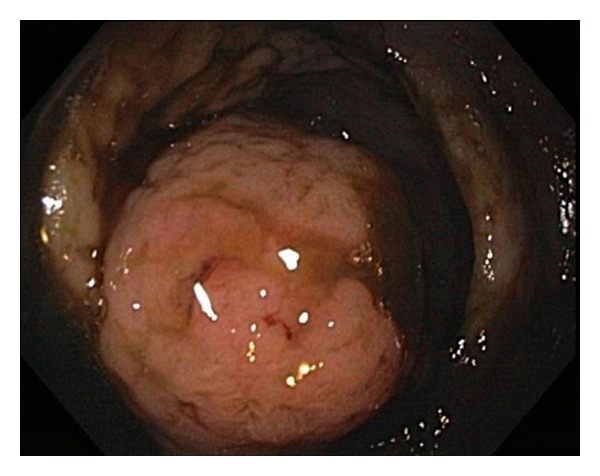
Colonoscopy image showing the polypoid cecal mass.

**Figure 2:**
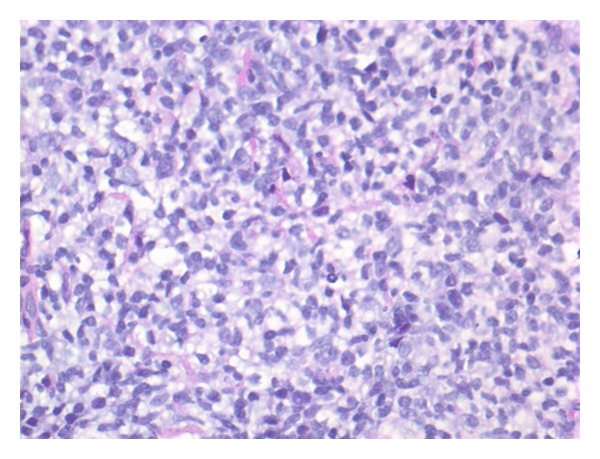
Diffuse proliferation of medium sized to large lymphoid cells, intermixed with few small lymphocytes.

**Figure 3:**
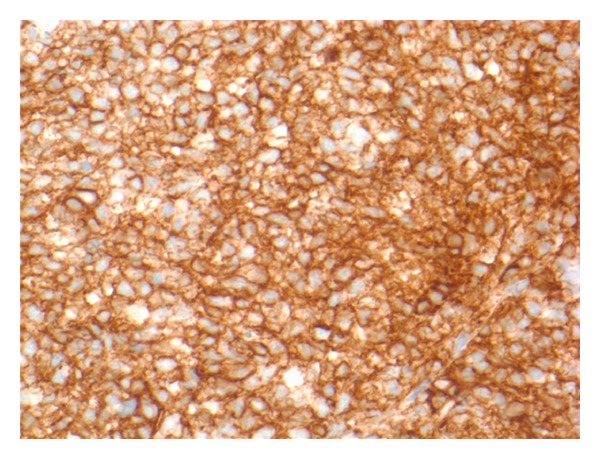
Tumor cells express CD20.

**Figure 4:**
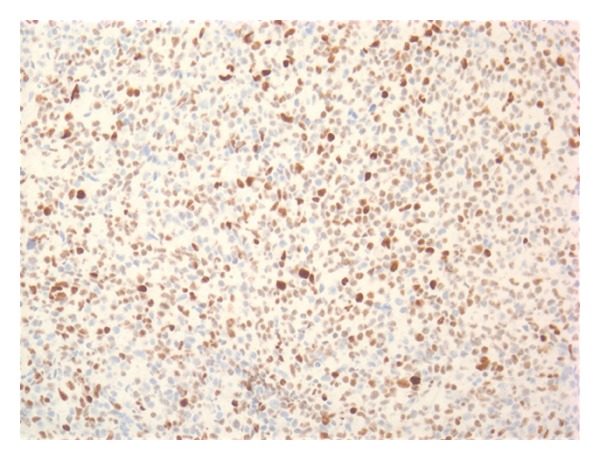
Most tumor cells are positive for BCL-6.

**Figure 5:**
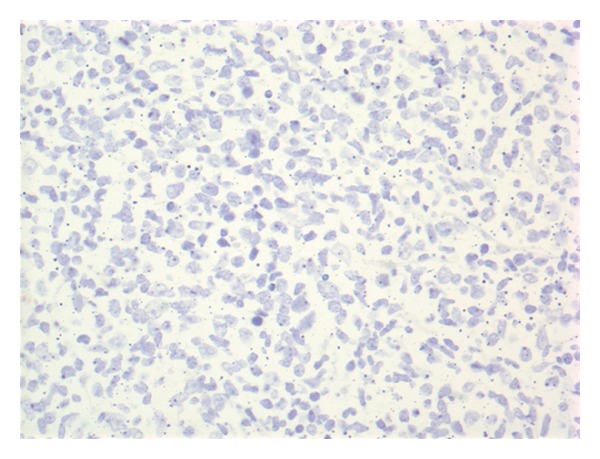
EBER *in situ* hybridization negative for EBV.

Given his anemia he underwent colonoscopy which revealed a 4.5 cm nodular and friable mass in the cecum (see Figure 1); biopsy diagnosed it as a DLBCL (see Figures 2, 3 and 4). Histologically, the mass showed a dense monomorphic infiltrate of abnormal lymphoid cells. Immunoprofile was typical of B-cell lymphoma (CD20 positive and negative for CD3, CD5, CD10, and cyclin D1). BCL6 showed patchy positivity suggestive of potential preexistent follicular lymphoma. EBV-encoded small RNA (EBER) *in situ* hybridization for EBV was negative (see Figure 5). Prognostically, the DLBCL had multiple favorable markers including germinal center origin (MUM-1 negative and BCL6 positive), low proliferative index (Ki-67 positive for only 25% of tumor cells), enhanced T-cell immune response (>60% CD3 positive cells), and low staining for P53 (only 10% of tumor cells). EBV blood level was ~1200 copies/mL; CMV blood level was <300 copies/mL. Prior EBV and CMV levels from 2010 were <250 and 300 copies/mL, respectively. Noted were the serum C3 < 5 mg/dL, C4 < 1 mg/dL, and RF > 2000 IU/mL at admission; CH50 was not checked. Computed tomography (CT) of his head, chest and abdomen, and pelvis was only relevant for enlarged mesenteric lymph nodes. A bone marrow biopsy on day 12 displayed no evidence for malignant infiltration. On day 12 he received his first dose of cyclophosphamide-hydroxyldaunorubicin-oncovin-prednisone (CHOP) chemotherapy.

His Cr level reached its nadir of 3.4 mg/dL on day 14. By day 22, his BUN and Cr values were 123 mmol/L and 4.6 mg/dL, respectively, and his clinical status warranted the use of hemodialysis (HD). By hospital day 29 he completed 6 courses of plasmapheresis, received a dose of rituximab to complement his initial one time CHOP chemotherapy, and his electrolytes had stabilized in the setting of good urinary output such that HD could be discontinued. He was discharged on a dose of tacrolimus 0.5 mg bid. 

At 2 months after hospitalization a repeat colonoscopy did not show any further evidence of lymphoma and EBV levels were <250 copies/mL. A CT scan at 3 months displayed decrease in size of his mesenteric lymphadenopathy, but he still had positive serum cryoprecipitate levels. At 4 months he had completed 6 cycles of CHOP-R therapy. Currently his serum Cr remains around 3 mg/dL and is being evaluated for renal transplant. He continues on tacrolimus 0.5 mg bid and prednisone 20 mg daily immunosuppression and valacyclovir for prophylaxis. All liver enzyme and INR values are normal.

## 3. Discussion

OLT has proven to be a successful therapy for fulminant liver failure and advanced chronic liver disease [1]. Nevertheless, given the necessary use of immunosuppressive therapies, such as tacrolimus, and the increasing survival rates of these patients, the frequency of PTLD has increased [3, 5]. PTLD comprises a spectrum of diseases and is highly associated EBV infection in 90% of documented cases. PTLD tends to occur at a median of 7–10 months post-OLT and the clinical presentation can range from typical “B-symptoms” of fevers and weakness to gastro-intestinal symptoms [3, 5, 6]. 

In the current report, a 53-year-old male with pre-transplant treated HCV and HCC developed a cecal 4.5 cm DLBCL with mesenteric nodal dissemination approximately 4 years after OLT complicated by cryoglobulin-induced AKI. Cryoglobulinemia in this case was more likely driven by the PTLD B cells than his HCV, the latter which was successfully treated prior to OLT. His PTLD and associated AKI were treated with CHOP-R chemotherapy and plasmapharesis, and the tacrolimus dosage was lowered by the time of discharge.

This patient's case brings up a number of interesting points. The presentation of PTLD is typically much earlier for OLT patients than as presented here, although this particular presentation is not unexpected either. His symptomatic anemia is also consistent with a gastrointestinal presentation of PTLD. Interesting is the negative EBV immunostain of the cecal mass but positive EBV blood levels which may indicate the poor sensitivity of the former and the high sensitivity of the later test. EBV-negative PTLD tends to occur later than EBV positive cases, is more likely to be monomorphic, such as in this case, and is more common in adults [7, 8]. 

Given the known reversibility of these PTLD lesions to cessation and/or reduction in immunosuppression therapies, his tacrolimus dose was reduced [2, 3]. Other options include switching to the non-calcineurin inhibitor, sirolimus, a mammalian target of rapamycin (mTOR) inhibitor, which has less renal toxicity [3]. Given the (1) long-term normal levels of his liver injury tests and the absence of any fibrosis or rejection on recent liver biopsy, (2) the duration after OLT and the known degree of chimerism that takes place vis a vis the host immune system and the allograft, and (3) his clinical tolerance of tacrolimus, dose reduction rather than switching to another medication seemed a sensible first maneuver. This method has produced satisfactory results in the author's (David H. Van Thiel) own experience and is similar to others [4]. It is rather interesting that historically his tacrolimus plasma levels were not simply within recommended ranges, but chronically quite lower (2-3 ng/mL in the preceding 3 years) with still excellent immunosuppression (chronically normal liver injury tests and biopsies). 

Furthermore, the presentation of a colonic PTLD renews concerns for the utility of colonoscopic surveillance after OLT. For instance, in patients with primary sclerosing cholangitis (PSC) and inflammatory bowel disease, the known intrinsic risk for colonic malignancy is quite real and thus colonoscopic surveillance every 1-2 years has been advocated regardless of OLT status. One study looked at otherwise healthy non-PSC OLT patients comparatively and their risk for colonic malignancy and has suggested that these patients follow standard screening and surveillance protocols similar to those without OLT [9]. Thus beyond the finding of alarm signs and symptoms, such as anemia seen in this presentation, accelerated rates of after OLT endoscopic surveillance are likely not cost-effective.

In summary, PTLD represents a not uncommon occurrence in OLT patients, and is highly correlated with EBV infection, and the duration and intensity of immunosuppressive therapies. This patient presented with a less common location, that is, the colon, of DLBCL, with features of symptomatic anemia and cryoglobulin-induced AKI, despite historically very low tacrolimus levels. This case highlights the concern and utility for further research into screening (serologically and/or endoscopically) for PTLD in OLT patients.

## Figures and Tables

**Table 1:** Key laboratory data during hospitalizations^∗^.

	10/12/11	2/17/12^*α*^	2/27/12	3/1/12^*∧*^	3/7/12^†^	3/9/12	3/16/12	Normal range
BUN (mg/dL)	33	63	88	92	129	123	99	8–21
Cr (mg/dL)	2.1	4.9	4.02	3.4	3.8	4.6	3.6	0.75–1.20
Alb (g/dL)	3.7	2.6	3.1	3.8	2.8	2.9	2.2	3.5–5.0
TB (mg/dL)	0.2	0.2	0.3	0.3	0.7	0.5	0.3	0.2–1.3
AP (U/L)	44	41	38	17	21	23	53	30–125
AST (U/L)	15	12	16	10	8	8	10	3–44
ALT (U/L)	9	6	7	6	7	7	9	0–40
INR (no units)	—	1.2	1.2	—	1.3	—	—	0.83–1.2
WBC (10^3^/*μ*L)	4.5	3.5	4.1	2.3	0.55	6.0	5.5	4.0–10.0
HGB (g/dL)	9.7	7.6	8.1	8.3	10.4	9.4	7.8	13.5–17.5
PLT (10^3^/*μ*L)	210	246	248	225	109	82	204	150–399
Tac (ng/mL)	6.6	3.1	—	3.7	4.3	6.6	3.0	No range

BUN: blood urea nitrogen; Cr: creatinine; alb: albumin; TB: total bilirubin; AP: Alkaline phosphatase; AST: aspartate aminotransferase; alanine aminotransferase; INR: international normalized ratio; WBC: white blood cells; HGB: hemoglobin; PLT: platelets; Tac: tacrolimus; —: no lab drawn that day. ^*α*^Day of admission; ^*∧*^2 days after chemotherapy; ^†^first day of hemodialysis.
